# Maternal Satisfaction on Delivery Service among Postnatal Mothers in a Government Hospital, Mid-Western Nepal

**DOI:** 10.1155/2018/4530161

**Published:** 2018-06-24

**Authors:** Asha Panth, Praveena Kafle

**Affiliations:** ^1^Nursing campus Nepalgunj, Institute of Medicine, Tribhuvan University, Nepal; ^2^LNGO: Unity for Sustainable Community Development (USCD), Bhairahawa, Nepal

## Abstract

**Background:**

Maternal satisfaction is one of the most frequently reported outcome measures for quality of care, and it needs to be addressed to improve the quality and efficiency of health care during pregnancy, childbirth, and puerperium to provide quality maternal-friendly services.

**Objective:**

To find out the maternal satisfaction on delivery service among postnatal mothers in a government hospital, Mid-Western Nepal.

**Method:**

A descriptive, cross-sectional study was conducted in maternity ward of Bheri Zonal Hospital, Nepal. A total of 178 purposively selected postnatal mothers were interviewed face-to-face using semistructured interview schedules. Analysis and interpretation of the findings were done with the help of descriptive and inferential statistics.

**Results:**

The study shows that majority (89.88%) of the mothers were satisfied with the delivery service. The level of satisfaction was higher in interpersonal and technical aspects (93.82%) of care than in informative aspects (91.57%) and health facility-related statements (91.01%). There was no statistically significant association between sociodemographic and obstetric characteristics and maternal satisfaction. Although insignificant, postnatal mothers who were illiterate were 2.710 times more likely to be satisfied than who were literate (*p*=0.475; OR = 2.710; CI = 0.343–21.4), also postnatal mothers up to primary level were 2.850 times more likely to be satisfied than secondary level and above (*p*=0.241; OR-2.850; CI 0.622–13.056). Also, in this study, postnatal mothers who were multiparous were 2.352 times more likely to be satisfied with the delivery service than primiparous (*p*=0.111; OR = 2.352; CI = 0.801–6.907). Majority (87.1%) of the mothers would like to receive delivery service next time in the same hospital.

**Conclusion:**

Majority of mothers were satisfied by the delivery service. Care givers need to fully understand the expectations the mothers have and provide care that is consistent with those expectations. The health system should be devised to increase maternal satisfaction in the health institution and provide maternal-friendly service.

## 1. Introduction

Quality of care is the degree to which maternal health services for individuals and populations increase the likelihood of timely and appropriate treatment for the purpose of achieving desired outcomes. The use of services and outcomes are the result not only of the provision of care but also of women's experience of that care. The quality of care received by mothers and babies in developing countries is often reported as poor [1]. Client satisfaction is an important indicator for assessment of the quality of care provided [2].

Assessment of satisfaction with maternity services is crucial and helps in future utilization of service [3, 4]. Understanding a woman's perspective and her needs during childbirth and addressing them as part of quality-improvement programme can make delivery care safe, affordable, and respectful [5].

Childbirth is a crucial experience in women's life as it has a substantial psychological, emotional, and physical impact. A positive experience in childbirth is important to the woman, infant's health and well-being, and mother-infant relationship. Furthermore, it is useful for the care providers to guarantee the best preparation, health service, and support to childbearing women [6]. The memories and experiences of childbirth remain with the woman throughout her life. Clearly, the support and care they receive during this period is critical [7, 8].

Institutional delivery as percentage of expected pregnancies has been in increasing trend from 44 to 50 percent over the last three consecutive years in Nepal, but the number of women attending 3 postnatal visits has been in declining trend. The coverage of the three postnatal visits is only 44% [9]. Thus, maternal satisfaction needs to be addressed to increase coverage.

A nationally representative facility-based survey conducted across 13 districts in Nepal by client exit interviews showed that most of the respondents were satisfied with (very satisfied and satisfied) care received at the facility (86%), provider's skills (85%), involvement in decision-making (77%), cleanliness (70%), information received (69%), and assured confidentiality (67%). The impact of rapid increase in institutional birth rate in Nepal on women's satisfaction and planned future utilization of services is less well known. The measures to improve client experience of maternity care in Nepal should focus on improvement in physical environment along with improving the attitude and communication skill of service providers with prompt response [10].

### 1.1. Conceptual Framework

**Figure 1:**
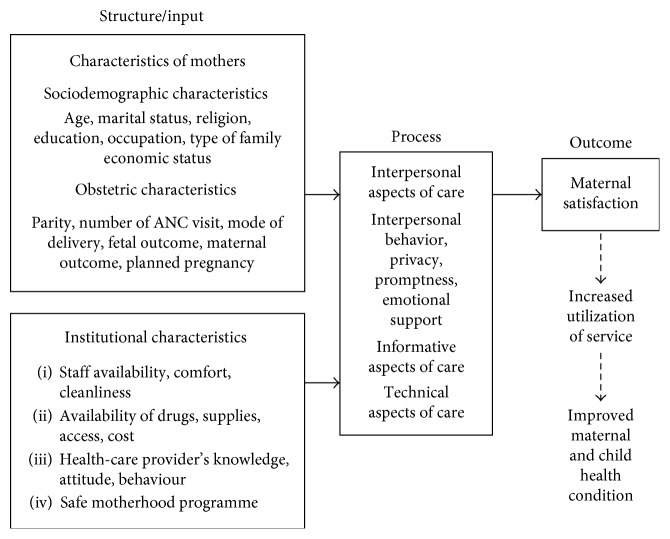
Maternal satisfaction based on the Donabedian model [11].

The conceptual framework was developed to understand the basis of study conducted. The framework was developed with the help of extensive literature review and was modified from the Donabedian model of assessment of quality of care which is shown in Figure 1. The Donabedian model is a conceptual model that provides a framework for examining health services and evaluating quality of health care. According to the model, information about quality of care can be drawn from three categories: “structure,” “process,” and “outcomes” [11].

Structure/input describes the context in which care is delivered, including hospital environment, knowledge, attitude and practice of staff, financing, and equipment, sociodemographics, and obstetric characteristics of mother. Process denotes the transactions between patients and providers throughout the delivery of healthcare. It includes interpersonal aspects of care, technical aspects of care, and informational aspects of care. Finally, outcomes contain all the effects of healthcare on patients, including changes to health status, patient satisfaction, and health-related quality of life.

## 2. Subjects and Methods

Descriptive cross-sectional study design based on quantitative approach was used in the study. The setting of the study area was maternity ward of Bheri Zonal hospital, Nepalgunj, which is implementing safe motherhood programme under the government of Nepal. This hospital serves as the referral centre for emergency obstetric care in mid-Western region of the country. It is also skilled birth attendant- (SBA-) training centre with the case load of around 450 deliveries per month. The study populations selected for the study were postnatal mothers who gave live birth at hospital and were about to be discharged from the hospital.

### 2.1. Sampling

The nonprobability purposive sampling technique was used. This was the most appropriate method of sampling as the judgment and deliberate effort were used to select postnatal mothers giving live birth at hospital and who were about to be discharged from hospital. During the data collection period of one month, one hundred and seventy-eight mothers were selected for the study.

A study conducted from a nationally representative facility-based survey across 13 districts in Nepal entitled “Women's Satisfaction of Maternity Care in Nepal and Its Correlation with Intended Future Utilization” showed prevalence of maternal satisfaction as 77% [10].

The sample size was calculated by using Cochran's formula [12]:

(1) $$\begin{array}{c} n_{o}=\frac{Z^{2}pq}{l^{2}}, \end{array}$$

with the desired precision of 5% (95% confidence limits at an allowable error of 5%), where *n*_o_*=* desired sample size; *Z* = the standard normal deviate (set for a 95% CI) = 1.96; *p* = the prevalence of maternal satisfaction = 0.77; *q* = 1 − *p* = 1 – 0.77 = 0.23; level of significance (*α*) = 5%; absolute allowable error (*l*) = 0.05; and *n*_o_ = (1.96)2∗(0.77)∗0.23/(0.05)2 = 270.65 = 271. For finite population, the sample size can be adjusted by using the following formula (*N*=450 (record of hospital)): *n*=*n*_o_/(1+(*n*_o_ − 1)/*N*) [13] = 169.37. To reduce nonresponse rate, additional 5% was taken, so 169.37 + 5% of 169.37 = 8.47. The final sample size was 177.82 = 178.

### 2.2. Exclusion Criteria

The selection was based on the exclusion criteria, that is, mothers who were admitted in the hospital after home delivery, having intrauterine fetal death, and those severely ill and not willing to participate in the study.

### 2.3. Instrumentation

A semistructured interview-based questionnaire on the basis of objectives and variable under study was developed by the researcher herself based on intensive literature review and consultation with experts.

The research instrument included the following parts:

 
*Part I* consists of questionnaires related to sociodemographic characteristics of mothers.
 
*Part II* consists of obstetric characteristics of mothers.
 
*Part III* consists of five-point Likert scale to access maternal satisfaction on delivery. There were four domains of care, that is, health facility-related statements (6 statements), interpersonal aspects of care (11 statements), informative aspects of care (10 statements), and technical aspects of care (9 statements). Score 5 was given for very satisfied, 4 for satisfied, 3 for neither satisfied nor dissatisfied, 2 for dissatisfied, 1 for very dissatisfied. Likewise, mean score less than or equal to 3 was considered as dissatisfied, whereas mean score greater than 3 was considered as satisfied. In other words, total score less than or equal to 108 was considered dissatisfied and above 108 was considered satisfied. Neutral score was considered dissatisfied, considering the respondents might be reluctant to express their negative feelings as they were interviewed in hospital setting.
 
*Part IV* consists of questionnaires related to mother's acceptance of service.

### 2.4. Validity and Reliability

The content validity of the test instrument was established by extensive literature review, consulting with advisors, subject matter experts, and nursing research faculty, as well as by peer review. First of all, the instrument was developed in English language then was translated into Nepali language and retranslated into English version to retain the same meaning. Opinion from the language expert was obtained for comprehensibility and simplicity of language during translation and back translation.

Cronbach's alpha was used for reliability analysis for satisfaction level. Result of Cronbach's alpha was 0.825 for overall satisfaction taking all statements.

Pretesting of the instrument was done in 10% of mothers admitted in maternity unit of Bheri Zonal hospital for clarity and comprehensibility of the tool. Those pretested mothers were excluded from the study. On the basis of pretesting, the instrument was revised and finalized for use in data collection.

### 2.5. Data Collection Procedure

Data were collected through face-to-face interview technique and the record review by the researcher herself after getting approval from the Research Committee, Maharajgunj Nursing campus, and Institutional Review Board (IRB) of Institute of Medicine. Permission was taken from the hospital authority with the submission of request letter from Maharajgunj Nursing Campus. Each respondent was briefed with the research objectives and informed written consent was obtained from the participants to ensure the right of the subject. Confidentiality was maintained throughout the study. Participants were given liberty to discontinue participating in the study if they wish. The participants were assured that the names will not be disclosed in the report and the information will be used for the study only. Precaution was taken throughout the study in every step to safeguard the right and welfare of all mothers in the study. The researcher provided needed information after completion of the interview.

The data were collected within the given time period of four weeks (February 27 to March 26, 2016). An exit interview, that is, the interview just before discharge, was taken with the mothers at their convenient time. The exit interview was selected to cover the maximum possible postnatal period during the hospital stay to assess overall maternal satisfaction on delivery service. About 20–25 minutes was taken to collect data from each respondent. At the end of interview, the collected forms were carefully checked for completeness and accuracy.

### 2.6. Data Analysis Procedure

Data were analyzed on the basis of research objectives and research questions. After collecting data, data were checked for accuracy, completeness, and consistency. The collected information was edited, coded, and entered in excel and afterwards transferred to SPSS version 16 for further analysis.

Analysis and interpretation of the findings were done with the help of descriptive statistics (frequency, percentage, mean, range, and standard deviation). In inferential statistics as Pearson's chi-square, Fisher's exact tests were used to test the association between the dependent and independent variables, and values ≤ 0.05 were taken for statistical significance at 95% confidence interval. Odds ratio was computed to find out the strength of association.

## 3. Results

Table 1 presents the sociodemographic characteristics of the postnatal mothers. Nearly half of the postnatal mothers (47.8%) were between 20 and 24 years of age, 18.5% were below 20, and only (2.2%) of the postnatal mothers were 35 years and above. The mean age of the postnatal mothers was 23.17 years with SD of 4.219 years and ranging from 16 to 37 years. Similarly, regarding ethnicity, one-third (33.7%) of the mothers belong to the Brahmin/Chhetri group followed by the Janajati group (23%). In regard to the type of family, most of the postnatal mothers (76.4%) were living in joint families. Likewise, all the postnatal mothers were married.

Table 2 shows that majority (87.1%) of postnatal mothers were literate and only 12.9% were illiterate. Among literate, more than half (52.3%) were secondary level and only 7.7% were bachelor level and above. Likewise, 62.9% of the postnatal mothers were homemakers, others included students. Similarly, majority of the postnatal mothers (82.6%) were Hindus followed by 13.5% Muslims. More than half of the postnatal mothers (52.8%) had household income enough for less than one year.

Table 3 shows the obstetric characteristics of mothers. More than half (54.5%) of the postnatal mothers were primiparous. Majority (80.43%) of the postnatal mothers had one child. Regarding sex of the recent baby, 56.7% were females and 43.3% were males. Likewise, majority of the postnatal mothers (93.3%) reported that their pregnancy was planned.

Table 4 shows the obstetric characteristics of postnatal mothers. Two-thirds of the postnatal mothers (66.3%) had visited antenatal clinic for four times. The postnatal mothers who had spontaneous vaginal delivery with intact perineum were 31.5% followed by caesarean section 28.7%. Similarly, majority (91%) of postnatal mothers did not have baby complications. Among the postnatal mothers who had baby complication, 25% had fetal distress and infection. Others included vomiting and swallowing difficulty. Likewise, only 2.2% had maternal complication. Among the postnatal mothers having maternal complication, all 3 had postpartum hemorrhage.

Table 5 shows maternal satisfaction related to health institution (6 statements), and majority (93.9%) were satisfied with transportation allowance and incentives among whom 43.3% were very satisfied, and 74% were satisfied with cleanliness of toilet among whom (8.4%) were very satisfied. Regarding service was free of cost, mean ± SD is 4.45 ± 5.99. The mean varies from 3.12 ± 1.103 to 4.37 ± 5.99.

Table 6 depicts the maternal satisfaction on items of interpersonal aspects of care (11 statements), higher percentage found in treated with dignity and respect (90.5%) among whom (37.1%) were very satisfied, and lowest in orientation (82.5%) among whom 44.9% were very satisfied. The mean varies from 4.17 ± 0.676 to 4.29 ± 0.724.

Table 7 reveals the maternal satisfaction on informative aspects of care (10 statements), in which highest percentage (85.4%) of postnatal mothers said they were satisfied with information received about the result of examination and the information about the freedom of position, and lowest percentage (44.4%) of postnatal mothers were satisfied with information provided about danger signs relating to mother and baby during postnatal period, among whom only (6.2%) were very satisfied. The mean varies from 3.39 ± 0.730 to 4.18 ± 0.753.

Table 8 shows technical aspects of maternal satisfaction (9 statements). Most of the postnatal mothers (95.5%) were satisfied with monitoring of blood pressure immediately after delivery, among whom 50.6% were very satisfied. Likewise, least of the postnatal mothers (53.4%) were satisfied with assisted in early ambulation, among which only 7.9% were very satisfied. The mean varies from 3.55 ± 0.729 to 4.46 ± 0.583.

Table 9 shows that majority of the postnatal mothers (93.82%) were satisfied and the minority (6.18%) of postnatal mothers were dissatisfied with interpersonal and technical aspects of care. Likewise, 91.57% were satisfied and 8.4% were dissatisfied with informative aspects of care. Similarly, 91.01% were satisfied and 8.99% were dissatisfied with health institution related statements.

Table 10 shows that majority (89.88%) of the postnatal mothers were satisfied and only 10.11% were dissatisfied with delivery service.

Table 11 shows that there is no statistically significant association between sociodemographic characteristics and maternal satisfaction. Although insignificant, postnatal mothers who were illiterate were 2.710 times more likely to be satisfied than who were literate (*p*=0.475; OR = 2.710; CI = 0.343–21.400); also, postnatal mothers up to primary level were 2.850 times more likely to be satisfied than secondary level and above (*p*=0.241; OR = 2.850; CI = 0.622–13.056).

Table 12 shows the association between obstetric characteristics and maternal satisfaction which is calculated by using chi-square test. No statistically significant association was found between the obstetric characteristics and maternal satisfaction. Although insignificant, those postnatal mothers who were multiparous were 2.352 times more likely to be satisfied with delivery service than primiparous (*p*=0.111; OR = 2.352; CI = 0.801–6.907).

Table 13 shows that majority (87.1%) of postnatal mothers would like and 4.5% would not like to receive delivery service next time in the same hospital. Majority (94.9%) would like to recommend the hospital to friends and relatives. Regarding the reason for choosing the hospital, more than two-thirds (68%) replied convenience and only 0.6% replied availability of operation facility.

In response to an open-ended question regarding suggestions for improving service, the postnatal mothers gave many suggestions that are presented in 11 themes in Table 14. The postnatal mothers who suggested to maintain cleanliness were 42.7% followed by to provide treatment properly were 22.5%. Likewise, 0.6% of the postnatal mothers suggested to take responsibility and not to scold. Also, 35.4% of the postnatal mothers replied that everything was good in the facility and required no improvement (no suggestions).

## 4. Discussion

Regarding statements related to health institution, 82.6% were satisfied with providing of necessary medicines and supplies, 92.1% were satisfied with the free-of-cost service, among whom 52.8% were very satisfied. Furthermore, the study shows 93.9% were satisfied with transportation allowance and incentives and 90.4% were satisfied with the prompt service. Also, only 64.6% were satisfied with the cleanliness of health institution and 41.5% were satisfies with the cleanliness and accessibility of toilet. Also, mean satisfaction score was lowest for cleanliness of the toilet. Consistent with this, the study conducted at Paropakar Maternity Hospital of Nepal showed that almost all (98.5%) of the respondents were satisfied with the free-of-cost service and transportation allowance (99.3%) and 78.2% were satisfied with general cleanliness of the facility. About 72.46% of the respondents were satisfied with the drug availability. Higher percentages were not satisfied with hospital environment (sanitation) [14]. Another study conducted in Ethiopia showed that 88.7% were satisfied with overall cleanliness of the health institution and 83.3% were satisfied with accessibility and cleanliness of toilets [4]. Similarly, inconsistent with the study, a nationally representative facility-based survey conducted across 13 districts in Nepal showed that maternal satisfaction was lowest for cleanliness of facilities [10]. Thus, hospitals of Nepal should maintain cleanliness of health institutions to increase maternal satisfaction.

Regarding statements related to interpersonal aspects of care, 85.4% were satisfied with warm welcome on admission, 82.5% were satisfied with orientation given, 84.3% were satisfied with maintenance of privacy, and 88.2% were satisfied with explanation given about treatment. In relation to involvement in decision-making, 85.4% were satisfied and 87.1% were satisfied with decision supported and respected. Consistent with the study, a study conducted in Ethiopia showed that 98% were satisfied with respect and assurance of privacy, 78.4% were satisfied with involvement in decision-making, and 92.3% were satisfied with given explanation about the treatment [4]. In contrast to the study, another study done in India showed that least satisfaction (48%) was found in the area related to orientation [15]. Another study done in Oromia showed that 78.9% of the delivering mothers were satisfied with respect and courtesy given from caregivers [16].

Regarding statements related to informative aspects of care, the study reveals that maternal satisfaction on receiving as much information as desired was 80.9%, result of examination and freedom of position 85.4%, benefits of diet 73.6%, state of newborn 82%, breast-feeding 81.4%, perineal and personal hygiene 76.4%, and information regarding baby care and immunization 62.9%. Only 46% were satisfied with information about postnatal follow-up visits, and 40.5% were satisfied with advice about danger signs. Thus, more information needs to be provided regarding postnatal visits and danger signs, but this area seems to be neglected. The study concludes that special attention should be focused on advices related to both postnatal mothers and their neonates. Postpartum women still required and needed guidance, more support, and assistance with baby care and their personal care. The study conducted in Tambacounda showed that maternal satisfaction increased when they received enough information on what to do in case of health problems, and the study showed that most of the mothers (93%) responded they received enough information as they wished [17].

Regarding technical aspects of care, majority (95.5%) were satisfied with monitoring of blood pressure immediately after delivery among whom 50.6% were very satisfied, and also 89.9% and 89.3% were satisfied with monitoring of fetal heart sound and the progress of labour, respectively. Similarly, 82.5% were satisfied with comfortable position and 69.6% with pain management. The study also shows maternal satisfaction on assisted in perineal care/wound care 60.2%, breast-feeding 54.5%, and early ambulation 53.4%, whereas in the study conducted in Ethiopia, pain control was the poorest source of satisfaction with 82% reporting dissatisfaction [18].

The study reveals that majority of the postnatal mothers (93.82%) were satisfied and 6.18% were dissatisfied with interpersonal and technical aspects of care. Likewise, 91.57% were satisfied and 8.4% were dissatisfied with informative aspects of care. Similarly, 91.01% were satisfied and 8.99% were dissatisfied with health institution related statements. The study reveals higher satisfaction in all aspects of care.

Regarding overall satisfaction, majority (89.88%) of the postnatal mothers were satisfied and only 10.11% were dissatisfied with the delivery service. Thus, the study shows that the mothers are satisfied with the service they have received. The study findings correspond to the study done in different countries. In consistent to the study, a nationally representative facility-based survey conducted across 13 districts in Nepal showed 86% were satisfied and very satisfied with the care received at the facility [10]. Similar study conducted in Oromia showed that the overall maternal satisfaction level with the delivery services rendered at the hospital was 80.7% [16]. In contrast to the finding, another hospital-based cross-sectional study done in Ethiopia showed that the proportion of mothers who were satisfied with delivery care was 61.9% [8].

### 4.1. Association between Sociodemographic Characteristics of Postnatal Mothers and Maternal Satisfaction

In the present study, there is no statistically significant association between sociodemographic characteristics and maternal satisfaction. This may be due to small sample size. This is consistent with the study done in India, which showed that age, religion, type of family, occupation, dietary pattern, and obstetrical score were not significant with maternal satisfaction at 0.05 level [15].

Although insignificant, postnatal mothers whose age was 25 and above were more likely to be satisfied with delivery service than below 25 (OR = 1.365; CI = 0.462–4.017), and homemakers were more likely to be satisfied than other occupations (OR = 1.807; CI = 0.679–4.810). This finding is consistent with the study done in Lebanon, which showed that patients who are older are more satisfied with the childbirth experience and unemployed mothers were more satisfied with the childbirth experience than employed mothers [2].

Also, in this study, postnatal mothers who were illiterate were 2.710 times more likely to be satisfied than who were literate (*p*=0.475; OR = 2.710; CI = 343–21.400); also, postnatal mothers up to primary level were 2.850 times more likely to be satisfied than secondary level and above (*p*=0.241; OR-2.850; CI 0.622–13.056). Literates were less satisfied with delivery service may be due to more expectation of quality service. These findings are consistent with the findings of the study done in Oromia, showing that respondents who had no higher education were more satisfied than those who had diploma and above [16]. In contrast to this, the study done in Lebanon showed that women who reached college or more were more satisfied with the childbirth experience than women with an education of high school or less [2].

### 4.2. Association between Obstetric Characteristics of Postnatal Mothers and Maternal Satisfaction

In this study, there is no statistically significant association between obstetric characteristics and maternal satisfaction. In contrast to this study, the study done in Oromia showed that there is positive and significant association between ANC follow-up, wanted (planned status of pregnancy), maternal and fetal outcome, and maternal satisfaction [16]. Although insignificant, those postnatal mothers who were multiparous were 2.352 times more likely to be satisfied with delivery service than primiparous (*p*=0.111; OR = 2.352; CI = 0.801–6.907). This finding is consistent with the study done in Lebanon that showed multiparous women were slightly more satisfied than primiparous [2].

### 4.3. Mothers' Acceptance of Service

The present study shows majority (87.1%) of postnatal mothers would like and 4.5% would not like to receive delivery service next time in the same hospital. Majority (94.9%) would like to recommend the hospital to friends and relatives. This suggests that the hospital has skilled professionals providing an acceptable quality of care. Similarly, the study done in Oromia showed that most of the delivering postnatal mothers were very likely to recommend the facility to friends and family and also for themselves (99.4% and 97.5%), respectively [16].

Regarding the reason for choosing the hospital, 70.78% replied convenience, followed by quality care 23%, 11.2% referred, and only 0.6% replied availability of operation facility. In this study, postnatal mothers chose the particular hospital on the grounds of convenience. Consistent with the study, the study conducted in India showed that majority (95%) replied convenience and 94% of postnatal mothers chose the particular hospital because of the availability of good doctors, and referred from other hospital 84%, whereas 33% of postnatal mothers selected the particular hospital because of quality care by the staff nurse [15].

Regarding the mothers' responses for improving service, the postnatal mothers who suggested maintaining cleanliness were 42.7% followed by postnatal mothers who suggested to provide treatment properly were 22.5%. Likewise, 0.6% of the postnatal mothers suggested to take responsibility and not to scold. Also, 35.4% of the postnatal mothers replied that everything was good in the facility and required no improvement (no suggestions). Thus, the hospital environment should be clean so that the postnatal mothers can be satisfied. Consistent with the study, a nationally representative survey on maternal satisfaction conducted in Nepal showed that respondents suggested maintaining clean/hygienic health facilities (42%). About 17% of maternity clients responded that everything was good in the facility and required no improvement [10].

## 5. Conclusions

On the basis of study findings, it is concluded that majority of the postnatal mothers are satisfied with the delivery service. The level of satisfaction was higher in interpersonal and technical aspects of care than in informative aspects and health facility-related statements. The higher the education level, the lower the level of maternal satisfaction, and multiparous are more likely to be satisfied with delivery service than primiparous. Most of the postnatal mothers would like to receive delivery service next time in the same hospital. More than two-thirds of the postnatal mothers chose the hospital due to convenience. Although the majority of postnatal mothers are satisfied by the delivery service, lack of dissatisfaction by a minority of postnatal mothers may result in a limited ability to engage in health facility, which further contributes to maternal mortality.

### 5.1. Recommendations

Further studies can take into consideration the findings and limitations of this study for better results. The study could be done in community setting where postnatal mothers could freely express their satisfaction regarding the service they have received.

Care givers need to fully understand the expectations that the mothers have for their care and provide care that is consistent with those expectations. The health system should be devised to increase maternal satisfaction in the health institution and provide maternal-friendly service.

## Figures and Tables

**Table 1:** Sociodemographic characteristics of postnatal mothers: age, ethnicity, and type of family.

Variable	Number (*n*=178)	Percentage
Age (in years)		
<20	33	18.5
20–24	85	47.8
25–29	42	23.6
30–34	14	7.9
Above 35	4	2.2
Mean age = 23.17		
SD = ±4.219 years		
Range = 16–37 years		
Ethnicity		
Brahmin/Chhetri	60	33.7
Janajati	41	23.0
Dalit	21	11.8
Madhesi	32	18
Muslims	24	13.5
Type of family		
Nuclear	42	23.6
Joint	136	76.4

**Table 2:** Sociodemographic characteristics of postnatal mothers: educational status, educational level, occupation, religion, and family income status.

Variable	Number (*n*=178)	Percentage
*Educational status (n*=178)		
Illiterate	23	12.9
Literate	155	87.1

*Educational level (n*=155)		
Just read and write	14	9.0
Primary level	26	16.8
Secondary level	81	52.3
Higher secondary level	22	14.2
Bachelor level and above	12	7.7

*Occupation (n*=178)		
Agriculture	29	16.3
Homemaker	112	62.9
Business	13	7.3
Service	10	5.6
Labour	2	1.1
Others	12	6.7

*Religion (n*=178)		
Hinduism	147	82.6
Buddhism	2	1.1
Islam	24	13.5
Christianity	5	2.8

*Family income status (n*=178)		
Enough for less than six months	10	5.6
Enough for less than one year	94	52.8
Enough for one year and surplus	74	41.6

**Table 3:** Obstetric characteristics of postnatal mothers: parity, number of children, sex of recent baby, and planned pregnancy.

Variable	Number (*n*=178)	Percentage
*Parity*		
Primiparous	97	54.5
Multipara	81	45.5

*Number of living children*		
One	97	80.43
Two and more	81	19.57

*Sex of recent baby*		
Male	80	44.94
Female	98	55.05

*Planned pregnancy*		
Yes	166	93.3
No	12	6.7

**Table 4:** Obstetric characteristics of postnatal mothers: number of ANC visits, mode of delivery, and complication.

Variable	Number (*n*=178)	Percentage
*Number of antenatal visits*		
Less than 4 times	32	18.0
Four times	118	66.3
More than 4 times	28	15.7

*Mode of present delivery*		
Spontaneous vaginal delivery with intact perineum	56	31.5
Spontaneous vaginal delivery with tear	41	23.0
Spontaneous vaginal delivery (SVD) with episiotomy	25	14.0
Assisted vaginal delivery (vacuum)	5	2.8
Caesarean section (C/S)	51	28.7

*Complications of newborn*		
Yes	16	9.0
No	162	91.0

*If yes, complications (n*=16)		
Fetal distress	4	25
Jaundice	2	12.5
Low birth weight	3	18.8
Infection	4	25
Others	3	18.8

*Complications of mother*		
Yes	3	1.68
No	175	98.31

*If yes, complications (n*=3)		
Postpartum hemorrhage	3	100

**Table 5:** Maternal satisfaction related to health institution.

Statements related to Health Institution	Number (percentage) (*n*=178)					
	VS (%)	S (%)	NSND (%)	D (%)	VD (%)	Mean ± SD
Provided with all necessary medicines and supplies	68 (38.2)	79 (44.4)	30 (16.9)	1 (0.6)	—	4.20 ± 0.732
Cleanliness of health institution	35 (19.7)	80 (44.9)	50 (28.1)	12 (6.7)	1 (0.6)	3.76 ± 0.864
Cleanliness and accessibility of toilet	15 (8.4)	59 (33.1)	53 (29.8)	35 (19.7)	16((9.0)	3.12 ± 0.103
Got service promptly	86 (48.3)	75 (42.1)	15 (8.4)	2 (1.1)	—	4.38 ± 0.688
Service was free of cost	94 (52.8)	70 (39.3)	14 (7.9)	—	—	4.45 ± 0.638
Provided with transportation allowance/incentives	77 (43.3)	90 (50.6)	11 (6.2)	—	—	4.37 ± 5.99

SD = standard deviation, VS = very satisfied, S = satisfied, NSND = neither satisfied nor dissatisfied, D = dissatisfied, and VD = very dissatisfied.

**Table 6:** Maternal satisfaction on interpersonal aspects of care.

Statements related to interpersonal aspects of care	Number (percentage) (*n*=178)					
	VS (%)	S (%)	NSND (%)	D (%)	VD (%)	Mean ± SD
Warm welcome on admission	79 (44.4)	73 (41)	25 (14.0)	1 (0.6)	—	4.29 ± 0.72
Orientation	80 (44.9)	67 (37.6)	27 (15.2)	4 (2.2)	—	4.25 ± 0.79
Privacy was maintained	66 (37.1)	84 (47.2)	27 (15.2)	—	1 (0.6)	4.21 ± 0.7
Not allowed to feel lonely	70 (39.3)	83 (46.6)	21 (11.8)	4 (2.2)	—	4.23 ± 0.74
Provided with emotional support	69 (38.3)	87 (48.9)	22 (12.4)	—	—	4.26 ± 0.67
Treated with dignity and respect	66 (37.1)	95 (53.4)	17 (9.6)	—	—	4.28 ± 0.63
Polite and helpful	70 (39.3)	84 (47.2)	22 (12.4)	2 (1.1)	—	4.25 ± 0.71
Attentive to needs and approached	66 (37.1)	90 (50.6)	22 (12.4)	—	—	4.25 ± 0.66
Explained about the treatment given and procedure	64 (36.0)	93 (52.2)	20 (11.2)	1 (0.6)	—	4.24 ± 0.66
Involved in decision-making	57 (32)	95 (53.4)	25 (14)	1 (0.6)	—	4.17 ± 0.68
Decision respected and supported	55 (30.9)	100 (56.2)	22 (12.4)	1 (0.6)	—	4.17 ± 0.65

SD = standard deviation, VS = very satisfied, S = satisfied, NSND = neither satisfied nor dissatisfied, D = dissatisfied, and VD = very dissatisfied.

**Table 7:** Maternal satisfaction on informative aspects of care.

Statements related to informative aspects of care	Number (percentage) (*n*=178)					
	VS (%)	S (%)	NSND (%)	D (%)	VD (%)	Mean ± SD
Gave as much information as desired	49 (27.5)	95 (53.4)	31 (17.4)	3 (1.7)	—	4.07 ± 0.72
Information about the results of examination	54 (30.3)	98 (55.1)	24 (13.5)	2 (1.1)	—	4.15 ± 0.61
Information about the freedom of position.	53 (29.8)	99 (55.6)	24 (13.5)	2(1.10	—	4.14 ± 0.68
Information about the benefits of diet	56 (31.5)	75(42.10)	41 (23.0)	6 (3.4)	—	4.02 ± 0.83
Information regarding the state of newborn after examination	57 (32.0)	89 (50.0)	30 (16.9)	2 (1.1)	—	4.13 ± 0.72
Information about breast-feeding	67 (37.6)	78 (43.8)	31 (17.4)	2 (1.1)	—	4.18 ± 0.75
Information about postnatal follow-up visits	15 (8.4)	67 (37.6)	81 (45.5)	14 (7.9)	1 (0.6)	3.46 ± 0.78
Advice about danger signs relating to mother and newborn baby during postnatal period	11 (6.2)	61 (34.3)	93 (52.2)	12 (6.7)	1 (0.6)	3.39 ± 0.73
Information regarding hygiene	54 (30.3)	82 (46.1)	42 (23.6)	—	—	4.07 ± 0.73
Information regarding baby care and immunization	31 (17.4)	81 (45.5)	58 (32.6)	8 (4.5)	—	3.76 ± 0.79

SD = standard deviation, VS = very satisfied, S = satisfied, NSND = neither satisfied nor dissatisfied, D = dissatisfied, and VD = very dissatisfied.

**Table 8:** Maternal satisfaction on technical aspects of care.

Statements related to technical aspects of care	Number (percentage) (*n*=178)					
	VS (%)	S (%)	NSSD (%)	D (%)	VD (%)	Mean ± SD
Provided with nonpharmacological methods of pain relief	25 (14)	99 (55.6)	46 (25.8)	8 (4.5)	—	3.79 ± 0.734
Monitored FHS regularly	87 (48.9)	73 (41)	18 (10.1)	—	—	4.39 ± 0.665
Monitored blood pressure immediately after delivery	90 (50.6)	80 (44.9)	8 (4.5)	—	—	4.46 ± 0.583
Monitored the progress of labour	83 (46.6)	76 (42.7)	17 (9.6)	2 (1.1)	—	4.35 ± 0.699
Assisted in maintaining personal hygiene	54 (30.3)	77 (43.3)	45 (25.3)	2 (1.1)	—	4.03 ± 0.77
Assisted in breast-feeding	28 (15.7)	69 (38.8)	72 (40.4)	9 (5.1)	—	3.65 ± 0.804
Kept in comfortable position	46 (25.8)	101 (56.7)	31 (17.4)	—	—	4.08 ± 0.654
Assisted in perineal care/wound care	17 (9.6)	90 (50.6)	66 (37.1)	5 (2.8)	—	3.67 ± 0.687
Assisted in early ambulation.	14 (7.9)	81 (45.5)	72 (40.4)	11 (6.2)	—	3.55 ± 0.729

SD = standard deviation, VS = very satisfied, S = satisfied, NSSD = neither satisfied nor dissatisfied, D = dissatisfied, and VD = very dissatisfied.

**Table 9:** Maternal satisfaction on four dimensions of delivery service.

Level of satisfaction (*n*=178)	Health institution-related statements (%)	Interpersonal aspects of care (%)	Informative aspects of care (%)	Technical aspects of care (%)
				
Satisfied	162 (91.01)	167 (93.82)	163 (91.57)	167 (93.82)
Dissatisfied	16 (8.99)	11 (6.18)	15 (8.426)	11 (6.18)

**Table 10:** Mothers' overall satisfaction level with delivery service.

Level of satisfaction	Number (*n*=178)	Percentage
Dissatisfied	18	10.11
Satisfied	160	89.89

**Table 11:** Association between sociodemographic characteristics of postnatal mothers and maternal satisfaction.

Satisfaction level (*n*=178)				
Variables	Satisfied, *N* (%)	Dissatisfied, *N* (%)	*p* value	Unadjusted OR (95% CI)
*Age*				
25 and above	55 (91.7)	5 (8.3)	0.575	1.365 (0.462–4.017)
Up to 25	105 (89.0)	13 (11.0)		1

*Ethnicity*				
Brahmin/Chhetri	54 (90.0)	6 (10.0)	0.972	1.019 (0.363–2.863)
Others	106 (89.8)	12 (10.2)		1

*Type of family*				
Nuclear	38 (90.5)	4 (9.5)	1.000^a^	1.090 (0.339–3.510)
Joint	122 (89.7)	14 (10.3)		1

*Educational status*				
Illiterate	22 (95.7)	1 (4.3)	0.475^a^	2.710 (0.343–21.400)
Literate	138 (89.0)	17 (11.0)		1

*Educational level*				
Up to primary	38 (95.0)	2 (5.0)	0.241^a^	2.850 (0.622–13.056)
Secondary and above	100 (87.0)	15 (13.0)		1

*Occupation*				
Homemaker	103 (92.0)	9 (8.0)	0.231	1.807 (0.679–4.810)
Others	5786.4)	9 (13.6)		1

*Religion*				
Non-Hindu	28 (90.3)	3 (9.7)	0.930^a^	1.061 (0.288–3.911)
Hindu	132 (89.8)	15 (10.2)		1

*Family income status*				
One year and surplus	67 (90.5)	7 (9.5)	0.807	1.132 (0.417–3.072)
Less than one year	93 (89.4)	11 (10.6)		1

Pearson's chi-square test; ^a^Fisher's exact test; ^*∗*^*p* value significant at <0.05 level; 1 = reference.

**Table 12:** Association between obstetric characteristics of postnatal mothers and maternal satisfaction.

Variables (*n*=178)	Satisfied, *N* (%)	Dissatisfied, *N* (%)	*p* value	Unadjusted OR (95% CI)
*Parity*				
Multiparous	76 (93.8)	5 (6.2)	0.111	2.352 (0.801–6.907)
Primiparous	84 (86.6)	13 (13.4)		1

*Planned pregnancy*				
Yes	149 (89.8)	17 (10.2)	1.000^a^	0.797 (0.097–6.557)
No	11 (91.7)	1 (8.3)		1

*Number of ANC*				
Less than 4 times	29 (90.6)	3 (9.4)	1.000^a^	1.107 (0.301–4.075)
4 and more than 4 times	131 (89.7)	15 (10.3)		1

*Mode of present delivery*				
Spontaneous vaginal delivery	115 (90.6)	12 (9.4)	0.643	1.278 (0.452–3.611)
Caesarean section	45 (88.2)	6 (11.8)		1

*Complication of newborn*				
Yes	15 (93.8)	1 (6.2)	0.591	1.759 (0.218–14.157)
No	145 (89.5)	17 (10.7)		1

Pearson's chi-square test; ^*∗*^*p* value significant at <0.05 level; ^a^Fisher's exact test; 1 = reference.

**Table 13:** Mothers' distribution according to acceptance of service.

Characteristics	Number (*n*=178)	Percentage
*Willingness to receive delivery service next time*		
Yes	155	87.1
No	8	4.5
Do not know	15	8.4

*Recommend the hospital*		
Yes	169	94.9
No	9	5.1

*Reason for choosing the hospital* ^*∗*^		
Convenience	126	70.78
Free of cost service	16	9.0
Quality care	41	23.0
Referred	20	11.2
Availability of operation facility	1	0.6

^*∗*^Multiple responses.

**Table 14:** Mother's recommendations for improving service.

Suggestions^*∗*^	Number (*n*=178)	Percentage
Maintain cleanliness	76	42.7
Provide treatment properly	40	22.5
Make more convenience	3	1.7
Work unitedly	3	1.7
Provide timely treatment	2	1.1
Provide information clearly	9	5.1
Companionship during delivery	3	1.7
Take responsibility	1	0.6
Not to scold	1	0.6
Make good conduct	5	2.8
No suggestions	63	35.4

^*∗*^Multiple responses.

## References

[1] Van den Broek N. R., Graham W. J. (2009). Quality of care for maternal and newborn health: the neglected agenda. *An International Journal of Obstetrics and Gynaecology*.

[2] Al Ahmar E., Tarraf S. (2014). Assessment of the socio-demographic factors associated with the satisfaction related to the childbirth experience. *Open Journal of Obstetrics and Gynecology*.

[3] Sawyer S., Ayers J., Abbott G., Gyte H., Rabe H., Duley L. (2013). Measures of satisfaction with care during labour and birth: a comparative review. *BMC Pregnancy and Childbirth*.

[4] Bitew K., Ayichiluhm M., Yimam K. (2015). Maternal satisfaction on delivery service and its associated factors among mothers who gave birth in public health facilities of Debre Markos Town, Northwest Ethiopia. *BioMed Research International*.

[5] Bhattacharyya S., Srivastava A., Avan B. I. (2013). Delivery should happen soon and my pain will be reduced: understanding women’s perception of good delivery care in India. *Global Health Action*.

[6] Bertucci V., Boffo M., Mannarini S. (2012). Assessing the perception of the childbirth experience in Italian women: a contribution to the adaptation of the childbirth perception questionnaire. *Midwifery*.

[7] Atiya K. M. (2016). Maternal satisfaction regarding quality of nursing care during labor and delivery in Sulaimani teaching hospital. *International Journal of Nursing and Midwifery*.

[8] Tayelgn A., Zegeye D. T., Kebede Y. (2011). Mothers’ satisfaction with referral hospital delivery service in Amhara Region, Ethiopia. *BMC pregnancy and Childbirth*.

[9] Annual Report (2016).

[10] Paudel Y. R., Mehata S., Paudel D. (2015). Women’s satisfaction of maternity care in Nepal and its correlation with intended future utilization. *International Journal of Reproductive Medicine*.

[11] Donabedian A. (1988). The quality of care: how can it be assessed. *JAMA*.

[12] Cochran W. G. (2007). *Sampling Techniques*.

[13] Panta P. P. (2012). *Biostatistics*.

[14] Shrestha B., Paneru D. P., Shrestha N., Dhimal B. (2010). Client’s satisfaction on maternity services at Paropakar maternity and women’s hospital. Kathmandu. *JHAS*.

[15] Varghese J., Rajagopal K. (2012). A study to evaluate the level of satisfaction perceived by postnatal mothers following nursing care in postnatal wards as expressed by themselves: pilot study. *Journal of Biology, Agriculture and Healthcare*.

[16] Amdemichael R., Tafa M., Fekadu H. (2014). Maternal satisfaction with the delivery services in Assela Hospital. *Gynecology and Obstetrics*.

[17] Oikawa M., Sonko A., Faye E. O., Ndiaye P., Diadhiou M., Kondo M. (2014). Assessment of maternal satisfaction with facility-based childbirth care in the rural region of Tambacouda, Senegal. *African Journal of Reproductive Health*.

[18] Melese T., Gebrehiwot Y., Bisetegne D., Habte D. (2014). Assessment of client satisfaction in labor and delivery services at a maternity referral hospital in Ethiopia. *Pan African Medical Journal*.

